# Construction of the First Russian Recombinant Live Attenuated Vaccine Strain and Evaluation of Its Protection Efficacy Against Two African Swine Fever Virus Heterologous Strains of Serotype 8

**DOI:** 10.3390/vaccines12121443

**Published:** 2024-12-21

**Authors:** Andrey Koltsov, Mikhail Sukher, Sergey Krutko, Sergey Belov, Alexey Korotin, Sofia Rudakova, Sergey Morgunov, Galina Koltsova

**Affiliations:** Federal Research Centre for Virology and Microbiology, Academician Bakoulov Street 1, 601125 Volginsky, Russiasergejjkrutko@gmail.com (S.K.); alescha.korotin@yandex.ru (A.K.);

**Keywords:** African swine fever, African swine fever virus, MGF360, MGF505, serogroup, serotype, genotype, live attenuated vaccine

## Abstract

**Background/Objectives:** The spread of African swine fever virus (ASFV) has led to major economic losses to pork worldwide. In Russia, there are no developed or registered vaccines against ASFV genotype II, which is associated with numerous ASFV outbreaks in populations of domestic pigs and wild boars in the country. **Methods:** We introduced deletions of the six MGF360 and MGF505 genes of the ASFV virulent Stavropol_01/08 strain, isolated in Russia in 2008. **Results:** We show here that this deletion did lead to full attenuation of the ASFV virulent Stavropol_01/08 strain. Animals intramuscularly inoculated with 10^4^ HAD_50_ of ΔMGF360/505_Stav developed a strong immune response and short period of viremia (at 3–7 days post-inoculation). Recombinant ΔMGF360/505_Stav strain provides complete protection of pigs against the ASFV parental Stavropol_01/08 strain (10^3^ HAD_50_). Therefore, in our experiment, we did not detect the genome of both the virulent and the recombinant strains in the blood and organs post-challenge with the Stavropol_01/08. In contrast, we found only partial protection (40%) of the ΔMGF360/505_Stav-immunized pigs against challenge with the ASFV heterologous Rhodesia strain. Additionally, the surviving animals had a prolonged fever, and their condition was depressed for most of the experiment. **Conclusions:** Thus, the ASFV recombinant ΔMGF360/505_Stav strain is the first live attenuated vaccine (LAV) in Russia that induces complete protection in pigs challenged with the highly virulent, epidemiologically relevant strains genotype II and serotype 8. However, this ASF LAV is not able to provide a high level of protection against other variants of serotype 8.

## 1. Introduction

African swine fever (ASF) is a viral hemorrhagic disease impacting domestic pigs, with a mortality rate that can reach up to 100% [1,2,3]. The disease’s causative agent, the African swine fever virus (ASFV), is a large DNA virus from the *Asfarviridae* family [4]. To date, several classifications of ASFV strains/isolates have been proposed. Thus, virus isolates have been divided into at least 24 genotypes based on the sequence of the B646L gene encoding the p72 protein [5,6,7,8,9]. At the same time, analysis of the entire encoded proteome allowed for the identification of seven distinct biotypes [8]. Another classification based on HAI typing and classical vaccination/challenge experiments divided ASFV isolates into at least eight discrete serogroups (serotypes), although there are probably more serogroups [10,11,12]. The results of serotype classification were in good agreement with the results of grouping based on the analysis of the EP402R and EP153R genes (encoding CD2v and C-type lectin proteins) [13]. It should be noted that the results of p72 genotyping and CD2v/lectin genotyping of ASFV isolates were often inconsistent [14].

The greatest diversity of ASFV genotypes and serotypes is found in Sub-Saharan Africa, where their transmission is sustained by a sylvatic cycle [15,16]. From 2007 to 2024, most outbreaks outside Africa (in Europe, Asia, America, and Oceania) were associated only with the genotype II ASFV [17,18]. In 2021, ASFV low-virulent p72 genotype I isolates were detected in the domestic pig population in China [19]. In 2023, highly virulent recombinant ASFV genotypes I and II were isolated in China and Vietnam [20,21,22].

In the 17 years since ASF was first diagnosed in the Caucasian region of Russia [23], ASFV outbreaks have been reported in various regions of Russia with enormous economic losses and serious effects on wild boar populations [24]. No vaccines against ASF are registered in Russia. Currently, two live attenuated vaccines (LAVs) against ASF are licensed and available in Vietnam—AVAC ASF live (AVAC VietNam, Vietnam) and NAVET-ASFVac (Navetco, Vietnam). Both of these vaccines are the ASFV recombinant genotype II strains with virulence-associated gene deletions [25,26,27,28]. In addition, several LAVs against ASF have been developed by researchers in different countries [27,29,30,31,32,33,34,35,36,37]. However, the use of live vaccines is associated with many risks, and various studies on their safety are needed, including in the field.

Different vaccine strategies for ASF have been evaluated, but the vaccines against ASF with full or partial protection are mainly based on field low-virulent ASFV strains [38,39,40,41], attenuated by consecutively passing in homologous or heterologous cell lines [12,42,43,44,45], and recombinant ASFV strains with the deletion of the virulence-associated genes [25,26,27,28,29,30,31,32,33,34,35,36,37]. Currently, editing the ASFV genome by homologous recombination or CRISPR/Cas9 technology for specific deletion of the virulence-associated genes seems to be the most promising method for the generation of ASF LAV vaccines.

Several LAV candidates obtained in different countries [25,31], as well as the licensed vaccine AVAC ASF live, were based on genetically similar strains of ASFV genotype II with a deletion of the three genes of the multigene family MGF360 and three genes of the multigene family MGF505. It should be noted that some LAV candidates had additional deletions of other genes [31]. These vaccine strains demonstrated a high level of protection against homologous infection. In addition, previously, we showed that the KK262 (Congo-a) vaccine strain serotype 2 revealed an 8.8 kb deletion in the left variable region of the genome, affecting five genes of the MGF360 and three genes of the MGF505, compared to the virulent homolog K49 [46]. Thus, it is obvious that the genes of multigene families MGF360/505 are associated with ASFV virulence, and this deletion does not affect the protective properties of deletion mutants.

In this study, we have successfully deleted three genes of the multigene family MGF360 (MGF360-12L, MGF360-13L, MGF360-14L) and three genes of the multigene family MGF505 (MGF505-2R and a partial deletion of MGF505-1R and MGF505-3R) from the genome of the ASFV virulent Stavropol_01/08 strain (genotype II, serotype 8). The recently developed recombinant ΔMGF360/505_Stav strain was created with the potential for use as an LAV against ASF and as a foundation for the production of other recombinant LAVs. Consequently, the assessment of its safety and efficacy against the parental strain became a pivotal objective of this study. The in vivo experiments demonstrated that the ΔMGF360/505_Stav strain is fully attenuated, exhibiting no adverse effects during the 30-day observation period and providing complete protection (100%) to immunized animals against the homologous virulent strain. We detected no or minimal viral genome in circulation in the blood and organs both post-immunization and post-challenge, which confirmed the new LAV candidate’s safety. In addition, we tested the protective properties of this recombinant strain against another ASFV virulent Rhodesia strain of serotype 8. Interestingly, this strain was assigned to the same serogroup as the Stavropol_01/08 strain based on HAI typing and CD2v/lectin genotyping, although based on the results of p72 genotyping, they were assigned to different genotypes [14]. We found only partial protection (40%) of the ΔMGF360/505_Stav-immunized pigs against challenge with the ASFV Rhodesia strain, and the surviving animals had a prolonged fever.

## 2. Materials and Methods

### 2.1. Viruses and Cell Cultures

Two virulent strains of serotype 8, Stavropol_01/08 (genotype II, GenBank accession number: JQ771686.1) and the reference strain Rhodesia (genotype VIII, GenBank accession number: KM609354.1), as well as reference strains of serotypes 1, 2, 3, and 4 (L57 (GenBank accession number: KM609344.1), K49 (Congo-v, GenBank accession number: MZ202520.1), M78 (GenBank accession number: KJ671548.1), and F32 (France-v, GenBank accession number: KJ671547.1), respectively) [12,13] were used in this study. The Russian ASFV Stavropol_01/08 strain was previously characterized and had been used to obtain deletion mutants of the ASFV [47].

DMEM/F-12 medium (PanEco, Moscow, Russia) was used to grow COS-1 cells as described by us previously [47]. Primary cultures of porcine macrophages were used for limiting dilutions, generation of viral stocks, titration of viral materials, and virus isolation. To prepare primary cultures of porcine macrophages from pig donor blood using Ficoll solution (PanEco, Russia), the standard protocol was used as described previously [47]. Virus titers were calculated based on the presence of hemadsorption in ASFV-infected cells and were expressed as 50% hemadsorption doses (HAD_50_) using the Reed–Muench method [48] as described previously [47].

All viruses and COS-1 cells were obtained from the collection of the FRCVM (Volginsky, Russia).

### 2.2. Animals

Specific pathogen-free piglets of the Large White pig breed, not vaccinated against any infections, were used as donors for blood collection. Animals were received from the Experimental Animal Preparation Sector of the FRCVM (Volginsky, Russia).

For the remaining experiments, clinically healthy animals (weight 15–20 kg) from Russian commercial farms were used. The animals were vaccinated against porcine circovirus type 2 (PCV2) using the vaccine Ingelvac CircoFLEX (Boehringer Ingelheim Animal Health, Ingelheim am Rhein, Germany). Inoculation with the ASFV strains was carried out no earlier than 3 weeks after immunization against PCV2.

The absence of swine pathogens in animals before the experiment procedures was confirmed by real-time PCR using commercial kits (VectorBest, Moscow, Russia) as described previously [47].

### 2.3. Plasmids and Cloning

DNA fragments for further molecular cloning were amplified using specific primers (DelMGF360/505_Larm_EcoRI_F_tatatagaattcttatctttgttcataatcaagaaaaatcc; DelMGF360/505_Larm_NsiI_R_ttttttatgcatctcccacgctaataaaagg; DelMGF360/505_Rarm_SalI_F_tttttgtcgactcctgtggacaggacccc; DelMGF360/505_Rarm_SphI_R_tatatagcatgcgcggccattttaatcagtttttcc) (Evrogen, Moscow, Russia). Generation of the recombination cassette based on the pUC57 vector (Thermo Scientific, Waltham, MA, USA) was performed as described previously [47]. The resulting recombination cassette contained two recombination arms and the reporter EGFP gene (under the control of the E184L gene promoter) located between them. The nucleotide sequence of the recombinant plasmid was verified by Sanger sequencing.

### 2.4. Construction of the Recombinant ΔMGF360/505_Stav Strain

The recombinant ΔMGF360/505_Stav strain with 6 gene deletion was constructed from the virulent ASFV Stavropol_01/08 strain by homologous recombination using a recombination cassette, as described previously [47,49]. Infected/transfected cells were incubated 5 days post-infection (dpi), then collected and stored at −50 °C.

To separate recombinant virus from wild-type virus, the method of limited dilutions in primary cultures of porcine macrophages was used and repeated at least 8 times. Selection of the recombinant ΔMGF360/505_Stav strain was based on the reporter fluorescence GFP protein using a ZOE fluorescent cell imager (Bio-Rad Laboratories, Hercules, CA, USA) and an Olympus CKX53 inverted microscope (Olympus, Shinjuku, Japan). The absence of “wild type” (parental strain) was confirmed using the pair of the MGF360-14L gene-specific primers (F_agaagacggggttcggatacag; R_gcaaatcctgaatatgggcttatacg) [50] by SYBR Green PCR as described previously [47].

### 2.5. Viral Replication Test In Vitro

A comparative analysis of in vitro replication in the primary culture of porcine macrophages of the parental strain and the recombinant ΔMGF360/505_Stav strain was carried out as described previously [51]. To confirm the obtained results, three independent experiments were conducted.

### 2.6. Animal Experiments In Vivo

Experiment 1. For initial safety studies of the deletion mutants, 6 crossbred piglets weighing 15–20 kg (mixed sex) were randomly divided into two groups. Each group of animals was kept in isolated rooms throughout the experiment. The acclimatization period for each group was 10 days. Group 1 consisted of four pigs that were intramuscularly (IM) inoculated with 1 mL of a 10⁴ HAD_50_ dose of the recombinant ΔMGF360/505_Stav strain. Group 2 served as the control group, with two animals remaining uninfected throughout the experiment (see Supplementary Materials, Figure S1A).

Clinical evaluation of ASF was carried out daily and was recorded as a clinical score, as described in [47,52]. The blood and serum samples collected from each animal at 0, 3, 5, 7, 14, and 21 dpi were used to assess viremia and the humoral immune response against ASF. At the end of the observation period (24 dpi), the animals were humanely euthanized for pathological evaluation and tissue (lung, liver, spleen, and mesenteric and submandibular lymph nodes) collection. All blood, serum, and tissue samples were stored at −80 °C for further analysis.

Experiment 2. The following stage of the work was to study the efficiency of the recombinant strain to protect animals against the homologous ASFV virulent Stavropol_01/08 strain. Twelve crossbred piglets weighing 15–20 kg (mixed sex) were divided into three groups. Piglets in group 1 (n = 5) were inoculated IM with 10^4^ HAD_50_ of the ASFV recombinant ΔMGF360/505_Stav strain, while piglets in group 2 (*n* = 5), serving as unvaccinated controls, were given 1 mL of PBS, with group 3 (*n* = 2) remaining uninfected throughout the experiment. At 28 dpi, all vaccinated piglets in group 1 and unvaccinated piglets from group 2 were challenged intramuscularly with 10^3^ HAD_50_ per pig of the parental Stavropol_01/08 strain (Supplementary Materials, Figure S1B).

All piglets from group 1 and group 2 were monitored daily after primary inoculation with the recombinant ΔMGF360/505_Stav strain or PBS, as well as after challenge with the virulent Stavropol_01/08 strain, and assigned a total clinical score based on assessment of clinical symptoms of 4 different categories, as described previously. The condition of animals from group 3 was assessed using the same criteria. The observation period was 30 days post-challenge (dpc).

The animals found dead or euthanized animals were subjected to a full postmortem analysis. Time to death, day-to-fever, and survival were recorded as described earlier [47,49]. As in Experiment 1, blood and serum samples collected before the challenge (at 0, 7, 14, 21, and 28 dpi) were analyzed to assess viremia and the humoral immune response against ASF. In addition, blood samples collected at 0, 3, 5, 7, 14, 21, and 28 dpc, as well as tissue samples (lung, liver, spleen, and mesenteric and submandibular lymph nodes) were tested for the ASFV DNA to assess viral load. All blood, serum, and tissue samples were stored at −80 °C for further analysis.

The blood samples collected at 7, 14, 21, and 28 dpc, as well as tissue samples (spleen and mesenteric and submandibular lymph nodes) from animals immunized with the ASFV recombinant ΔMGF360/505_Stav strain, were used for virus isolation by three consecutive passages in a primary culture of porcine macrophages.

Experiment 3. To evaluate the effectiveness of the recombinant strain to protect animals from infection with the heterologous virulent Rhodesia strain, which was also assigned to serotype 8, we repeated Experiment 2, but used the ASFV Rhodesia strain for the challenge. Thus, ten crossbred piglets weighing 15–20 kg (mixed sex) were divided into three groups. Piglets in group 1 (*n* = 5) were inoculated IM with 10^4^ HAD_50_ of the ASFV recombinant ΔMGF360/505_Stav strain, while piglets in group 2 (*n* = 3) serving as unvaccinated controls were given 1 mL of PBS, with group 3 (*n* = 2) remaining uninfected throughout the experiment. At 28 dpi, all vaccinated piglets in group 1 and unvaccinated piglets in group 2 were challenged intramuscularly with 10^3^ HAD_50_ per pig of the ASFV Rhodesia strain (Supplementary Materials, Figure S1C).

All experimental procedures were carried out similarly to Experiment 2, and the observation period was also 30 dpc.

### 2.7. Viral DNA Extraction and Quantitative PCR (qPCR)

To assess viral load in the blood and organ samples, B646L gene-based qPCR assays were used, as described previously [47]. To confirm the quality of viral DNA extraction, all samples were tested using beta-actin gene-specific primers and probes (internal PCR control (IPC)) [53,54].

DNA from blood samples was isolated using the ExtractDNA Blood kit (Evrogen, Moscow, Russia) according to the manufacturer’s instructions. ASFV DNA from tissue samples of infected animals was isolated using an automated magnetic bead-based KingFisher Flex System (Thermo Fisher, Waltham, MA, USA) and kit Magno-sorb (AmpliSens, Moscow, Russia) according to the manufacturer’s instructions.

### 2.8. ELISA and ELISPOT

For the detection of ASFV-specific antibodies, the p32, p62, and p72-antibody-specific IDScreen ASFV Indirect kit (IDVet, Grabels, France) was used according to the manufacturer’s instructions. Results are expressed as S/P% and the values were calculated according to the manufacturer’s instructions and as described in [47].

To assess the cellular response in immunized pigs, the Enzyme-Linked ImmunoSpot (ELISPOT) method was used. Detection of porcine peripheral blood mononuclear cells (PBMC) producing interferon gamma (IFN-γ) has been performed using a commercial Pig IFN-γ Single-Color ELISPOT kit (Cellular Technology Limited, Shaker Heights, OH, USA) according to the manufacturer’s instruction. The PBMCs of pigs were isolated from blood collected at 26 dpi, as described earlier [55]. The PBMCs were stimulated with ASFV antigen (ASFV, Stavropol_01/08 strain), Concanavalin A (ConA, positive control), medium (Neg, negative control). Points were counted using an Olympus CKX53 inverted microscope (Olympus, Shinjuku, Japan).

### 2.9. Hemadsorption Inhibition (HAI) Assays

Hemadsorption inhibition (HAI) assays were performed in swine macrophages using the ASFV parental Stavropol_01/08 strain and the ASFV recombinant ΔMGF360/505_Stav strain, hyper-immune reference antisera (serotypes 1, 2, 3, 4, 8), as well as data reference strain serotypes, as described previously [13,49]. The results of the HAI assay were recorded after 16–20 h by microscopy using an Olympus CKX53 inverted microscope (Olympus, Shinjuku, Japan).

### 2.10. Illumina Sequencing and Analysis

The total ASFV genomic DNA was extracted from the primary swine macrophage cells infected with the recombinant ΔMGF360/505_Stav strain or the parental Stavropol_01/08 strain, and used for whole-genome sequencing, as described previously [46]. Sequence alignments were generated using ClustalW algorithm.

### 2.11. Statistical Analysis

All Statistical analyses were performed using GraphPad Prism software version 8.0.1 (GraphPad Software, San Diego, CA, USA), as described previously. Briefly, two tests (the Mantel–Cox and the Gehan–Breslow–Wilcoxon) were used to analyze survival curves, while a one-way analysis of variance (ANOVA) was used to evaluate data on viral loads and the number of points in ELISPOT. The normality of the data was tested using the Shapiro–Wilk test and Kolmogorov–Smirnov test. Comparisons with a *p* value of <0.05 were considered statistically significant, and the *p* value in the figures was marked with asterisks (* *p* < 0.05; ** *p* < 0.01; *** *p* < 0.001; **** *p* < 0.0001). Statistically insignificant comparisons were marked in the figures as “ns”.

## 3. Results

### 3.1. Generation of the ASFV Recombinant ΔMGF360/505_Stav Strain

**Figure 1:**
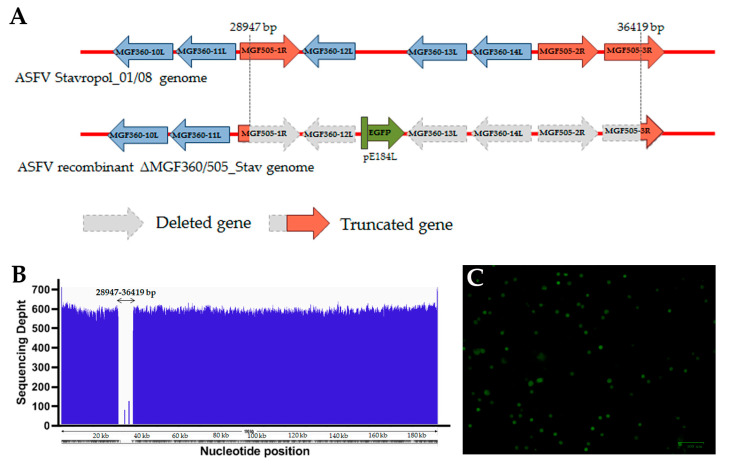
Generation of the ASFV recombinant ΔMGF360/505_Stav strain. (**A**) Scheme of the location of the MGF360/MGF505 gene deletion in the ASFV Stavropol_01/08 genome. (**B**) The recombinant ΔMGF360/505_Stav genome coverage plot using Illumina reads mapped to the ASFV Stavropol_01/08 genome. (**C**) Fluorescence microscopy of the primary culture of porcine macrophages infected with the ASFV recombinant ΔMGF360/505_Stav strain is shown. Scale bar indicates 100 μm.

In order to generate a new recombinant virus with a deletion of the three genes of the multigene family MGF360 (MGF360-12L, MGF360-13L, MGF360-14L) and three genes of the multigene family MGF505 (MGF505-2R and a partial deletion of MGF505-1R and MGF505-3R) with potential use as LAV, homologous recombination with a recombination cassette and the ASFV parental strain was used in this study. The parental virus was the ASFV virulent Stavropol_01/08 strain (genotype II, serotype 8), which is genetically very close to the ASFV Georgia 2007/1 strain (Gen-Bank NC_044959.2). The recombination cassette was based on the plasmid vector pUC57 and included the EGFP reporter gene with the E184L gene promoter and two recombination arms (Larm and Rarm) (Figure 1A, Supplementary Materials, Figure S2). COS-1 cells were used for infection with the ASFV parental strain and subsequent transfection with the recombination cassette. The primary macrophages were used for the selection of the ASFV recombinant ΔMGF360/505_Stav strain by seven rounds of limited dilution. The EGFP fluorescent protein signal was used as a selectable marker (Figure 1C).

The MGF360-14L gene sequence was used as a marker to detect the presence of the “wild type” (the ASFV virulent Stavropol_01/08 strain) by the SYBR Green PCR using primers complementary to this region. The absence of the “wild type” in the viral stock of the recombinant ΔMGF360/505_Stav strain was confirmed. Additionally, replacement of six MGF360/MGF505 genes with the EGFP gene was confirmed by amplification of the recombination site and the reporter gene (Supplementary Materials, Figure S2).

The accuracy of the genetic modifications introduced in the ASFV recombinant ΔMGF360/505_Stav strain genome, as well as possible additional nucleotide changes associated with passaging in the primary culture of porcine macrophages, were evaluated by sequencing the whole virus genome via next-generation sequencing (NGS). The full genome analysis confirmed the accuracy of the introduced modifications (Figure 1B). We found only four additional mutations in the ASFV recombinant ΔMGF360/505_Stav strain genome compared to the genome of the ASFV parental Stavropol_01/08 strain, which were insertions or deletions in the poly(C) or poly(G) regions, which could represent sequencing errors due to the complexity of analyzing these genomic regions.

### 3.2. Comparative Analysis of In Vitro Growth of the Recombinant ΔMGF360/505_Stav Strain and the Parental Stavropol_01/08 Strain in a Primary Culture of Porcine Macrophages

The recombinant ΔMGF360/505_Stav strain reached titers of 6–7 log10 HAD_50_/mL in macrophages. Similar titers for the parental strain were noted by us previously [47]. We also found no differences in the hemadsorption pattern of the ASFV recombinant ΔMGF360/505_Stav strain compared to the parental strain (Supplementary Materials, Figure S3A,B). Thus, the ASFV recombinant ΔMGF360/505_Stav strain and the ASFV parental Stavropol_01/08 strain exhibited similar replication and hemadsorption characteristics in macrophages.

**Figure 2:**
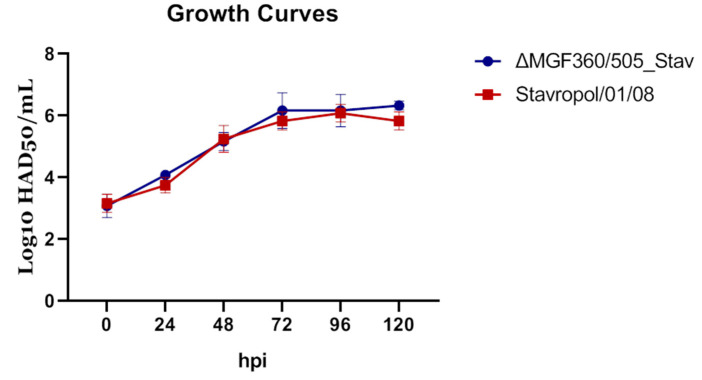
Comparative analysis of in vitro replication of the recombinant ΔMGF360/505_Stav strain (blue line) and the parental Stavropol_01/08 virus (red line) strain. The data are presented as the mean virus titer (expressed in log10 HAD_50_/mL) ± standard deviation (SD) at each point (0, 24, 48, 72, 96, and 120 h post-infection (hpi)).

To conduct a more detailed analysis of the replication of the recombinant strain, in vitro multistage growth curves of viruses were constructed, as described previously [47]. The ASFV parental Stavropol_01/08 or the recombinant ΔMGF360/505_Stav strains with a multiplicity of infection (MOI) of 0.1 were used to inoculate the cells. The ASFV replication was quantified by titration of infected cell samples collected at six time points (at 0, 24, 48, 72, 96, and 120 h post-infection (hpi)). Titration of these samples was carried out in primary cultures of porcine macrophages, and the titer was expressed as log10 HAD_50_/mL. The values of three independent experiments were used for analysis. As shown in Figure 2, the recombinant ΔMGF360/505_Stav strain showed growth similar to that of the parental strain. Therefore, we hypothesized that the recombinant ΔMGF360/505_Stav strain would be able to replicate efficiently not only in vitro, but also in vivo during infection in pigs, to mount an effective humoral and T-cell immune response.

### 3.3. HAI Serological Specificity of the Recombinant ΔMGF360/505_Stav Strain

As expected, deletion of the genes of the multigene families MGF360 and MGF505 did not lead to a change in HAI serological specificity. Thus, hemadsorption of both the parental Stavropol01/08 strain and the recombinant ΔMGF360/505_Stav strain was inhibited by the addition of serotype 8 reference antisera. In contrast, reference antisera of serotypes 1, 2, 3, and 4 did not inhibit hemadsorption of the Stavropol01/08 and the ΔMGF360/505_Stav strains (Supplementary Materials, Table S1). It should be noted that the ASFV HAI serological specificity is associated with the C-type lectin/CD2v locus (EP153R/EP402R genes) [13], and the sequences of these genes were identical in the Stavropol01/08 and the ΔMGF360/505_Stav strain genomes, so the results of the HAI serological specificity study were expected.

### 3.4. Study of the Safety and Immunogenicity of the Recombinant ΔMGF360/505_Stav Strain

To evaluate the replication of the recombinant ΔMGF360/505_Stav strain in the pig and to prove the safety of this strain, intramuscular inoculation with the recombinant strain (10^4^ HAD_50_/animal dose) was performed (Experiment 1, *n* = 4). The reverse titration of the viral material utilized for animal infection yielded confirmation that all pigs received an inoculum with a titer of 10⁴ HAD_50_, which aligns with the anticipated concentration.

**Figure 3:**
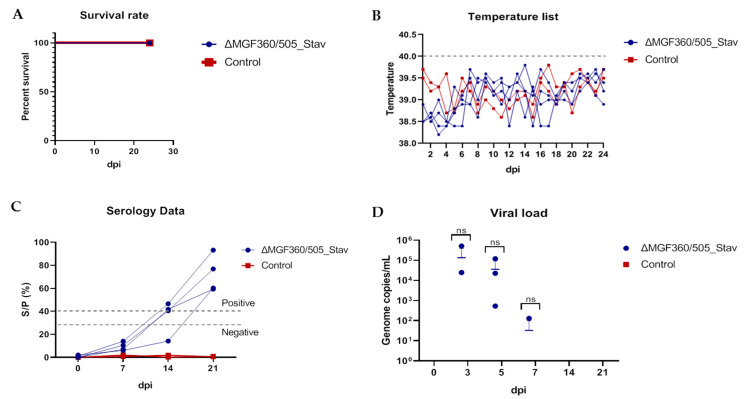
The safety and immunogenicity profile study of the ASFV recombinant ΔMGF360/505_Stav strain when inoculating pigs with 10^4^ HAD_50_ per animal. The lethality kinetics (**A**), body temperatures (**B**), humoral immune response (**C**), daily viral loads in blood (**D**) in the pigs inoculated with the ASFV recombinant ΔMGF360/505_Stav strain (blue line) and control non-inoculated animals (red line). (**B**) The fever cutoff (40 °C) is marked as a gray dashed line. (**C**) The dashed lines represent the cut-off value, above which values were considered positive, below which were considered negative, and between them, there was a gray area where the values were considered doubtful. Data on body temperature, antibody response, and viral load are presented as individual values for each animal. dpi—days post-infection. ns—*p* > 0.05.

Body temperature and other ASF clinical signs were monitored for 24 dpi. To assess the antibody response against ASF, viremia, and virus replication, blood, serum, and tissue samples were collected and analyzed. All animals inoculated with the ΔMGF360/505_Stav strain, as well as control animals (non-inoculated with ASFV), were alive until the end of the observation period (Figure 3A). The inoculated pigs did not show clinical signs of disease, and the body temperature of the animals remained below what was considered a fever (40.1 °C) (Figure 3B).

Viremia was analyzed in both ASFV-inoculated and ASFV-non-inoculated animals. As shown in Figure 3D and Table S2, in ASFV-inoculated animals, viremia was detected in three pigs (#1.1 at 3–7 dpi; #1.2 at 3–5 dpi; #1.3 at 5 dpi) and remained undetectable in one of the animals. Low levels of viremia (1.3 × 10^2^−5.11 × 10^5^ the ASFV genome copies/mL) were observed at early points post-inoculation, whereas viral DNA was not detected at later times. Furthermore, the ASFV genome was not detected in the organs of the inoculated animals that were euthanized humanely at 24 dpi (see Supplementary Materials, Table S2). As anticipated, no viral genome was detected in the ASFV-non-inoculated animals (Figure 3D, Supplementary Materials, Table S2).

Serum samples collected at 0, 7, 14, and 21 dpi were tested for the presence of antiASFV antibodies by ELISA. We demonstrated that all sera sampled from ASFV-inoculated animals at 0 and 7 dpi, as well as the serum sample from pig #1.3 at 14 dpi, were negative. The sera of ΔMGF360/505_Stav-inoculated pigs were found to be positive at 14 (except animal #1.3) and 21 dpi (Figure 3C, Supplementary Materials, Table S2). All sera from control uninfected animals were negative (Figure 3C, Supplementary Materials, Table S2).

These data reveal that the recombinant ΔMGF360/505_Stav strain is attenuated in vivo. Although the recombinant ΔMGF360/505_Stav strains replicated in vivo, thereby inducing the development of a humoral immune response, we did not observe any ASF-specific clinical signs post-inoculation, which indicated the safety of the obtained recombinant ΔMGF360/505_Stav strain.

### 3.5. Evaluation of the Efficiency of Immunization with the Recombinant ΔMGF360/505_Stav Strain for Protection Against the ASFV Parental Stavropol_01/08 Strain

To evaluate the efficiency of the recombinant strain to protect animals against the homologous virulent Stavropol_01/08 strain, three groups of pigs (*n* = 12) were used in Experiment 2. Piglets in group 1 (*n* = 5) were inoculated intramuscularly with 10^4^ HAD_50_ of the ASFV recombinant ΔMGF360/505_Stav strain. The reverse titration demonstrated that all pigs were inoculated with material containing 10^3.5^ HAD_50_, which was 0.5 log HAD_50_ lower than in Experiment 1. Piglets in group 2 (*n* = 5), as well as animals in group 3 (*n* = 2), were inoculated with 1 mL of PBS per animal.

No animals from any of the three groups demonstrated fever or any other clinical signs (Supplementary Materials, Figure S4A,B). We also did not detect ASFV DNA in in blood samples of the ASFV-inoculated or the ASFV-non-inoculated animals collected at 0, 7, 14, 21, and 28 dpi. It is possible that viremia in animals from group 1 occurred at earlier time points when samples were not collected.

**Figure 4:**
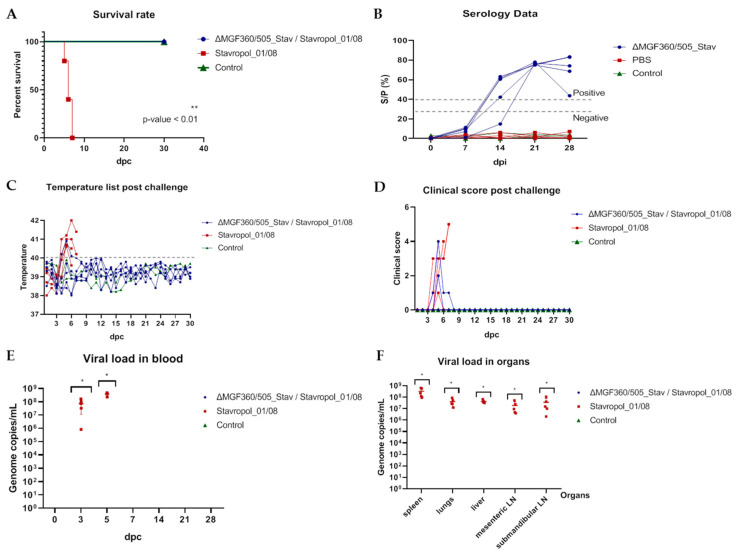
The efficiency of immunization with the ASFV recombinant ΔMGF360/505_Stav strain against the ASFV virulent parental Stavropol_01/08 strain when pigs were immunized with 10^4^ HAD_50_ per animal and then challenged with 10^3^ HAD_50_ per animal of the ASFV homologous Stavropol_01/08 strain (Experiment 2). The lethality kinetics (**A**), humoral immune response (**B**), body temperatures post-challenge (**C**), clinical signs post-challenge (**D**), daily viral loads in blood post-challenge (**E**), and post-mortem viral loads in organs (**F**) in pigs immunized with the ASFV recombinant ΔMGF360/505_Stav strain, infected with the Stavropol_01/08 strain (blue line), non-immunized animals, infected with the Stavropol_01/08 strain (red line), and control non-inoculated animals (green line). (**B**) The dashed lines represent the cut-off value, above which values were considered positive, below which were considered negative, and between them, there was a gray area where the values were considered doubtful. (**C**) The fever cutoff (40 °C) is marked as a gray dashed line. Data on body temperature, clinical assessment, antibody response, and viral load are presented as individual values for each animal. dpi—days post-immunization; dpc—days post-challenge. * *p* < 0.05.

Serum samples collected at 0, 7, 14, 21, and 28 dpi were tested by commercial ELISA, as described previously. The sera of the pigs inoculated with the ASFV recombinant strain were found to be positive at 14 (except animal #1.4), 21, and 28 dpi (Figure 4B, Supplementary Materials, Table S3). The remaining sera samples, including sera sampled from ASFV-inoculated animals at 0 and 7 dpi (group 1), and all sera from uninfected animals from groups 2 and 3 were negative (Figure 4B, Supplementary Materials, Table S3).

ELISPOT assay results showed that PBMCs from all pigs immunized with the recombinant strain produced IFN-γ after stimulation by the parental Stavropol_01/08 strain and also after stimulation by Concanavalin A (positive control). The numbers of spot-forming cells from immunized pigs were significantly higher than the cells from non-immunized pigs (Supplementary Materials, Figure S5A, Table S3). The numbers of spot-forming cells from immunized pigs stimulated with medium (negative control) were comparable to the numbers of spot-forming cells from non-immunized pigs (Supplementary Materials, Figure S5A, Table S3).

At 28 dpi, all piglets in group 1 and group 2 (positive control) were challenged intramuscularly with 10^3^ HAD_50_ per pig of the parental Stavropol_01/08 strain. Animals in group 3 (negative control) remained uninfected throughout the experiment.

In group 2 (positive control), mortality reached 100% by day 7 pc. (Figure 4A, Supplementary Materials, Tables S3 and S5). The incubation period was estimated as 2–4 days. The onset of fever in animals in this group was noted at 3–4 dpc (Figure 4C, Supplementary Materials, Tables S3 and S5). In contrast, in immunized animals in group 1, only minor clinical signs were noted after the challenge with the parental Stavropol_01/08 strain. Thus, one piglet (#1.1) did not have fever but had a slight apathy at 4 dpc (Figure 4B). Animal #1.2 had fever from 3 to 5 dpc (40.1–40.9 °C) but had no other serious clinical signs (Figure 4C,D, Supplementary Materials, Table S3). The other three animals, immunized with the recombinant strain, were completely clinically healthy after the challenge (Figure 4C,D, Supplementary Materials, Table S3). All the immunized animals, as well as control animals (non-inoculated with ASFV) were alive until the end of the observation period (30 dpc) (Figure 4A, Supplementary Materials, Table S3).

Regarding ASFV genome detection, viral DNA was not detectable in the blood or organs of immunized animals (Figure 4E,F, Supplementary Materials, Table S3). To confirm the absence of virus in these samples, we used it for virus isolation in the primary culture of porcine macrophages. Three consecutive passages failed to isolate the virus from any blood or organ samples from immunized animals.

In contrast, group 2 blood samples collected from 3 to 5 dpc, as well as organ samples, contained high numbers of ASFV genome copies (8.19 × 10^5^–6.33 × 10^8^ the ASFV genome copies/mL) (Figure 4E,F Supplementary Materials, Table S3). As expected, no viral genome was detected in the blood and organs of the ASFV non-inoculated animals (Figure 4E,F, Supplementary Materials, Table S3).

### 3.6. Evaluation of the Efficacy of Immunization with the Recombinant ΔMGF360/505_Stav Strain for Protection Against the ASFV Rhodesia Strain

Due to the high efficacy of the ASFV recombinant ΔMGF360/505_Stav strain in protecting against the ASFV homologous virulent Stavropol_01/08 strain, the next step of the study was to evaluate the efficacy of this strain against the heterologous strain Rhodesia, which is in same serogroup as the Stavropol_01/08 strain. Similar to Experiment 2, three groups of pigs (*n* = 10) were used in Experiment 3. Piglets in group 1 (*n* = 5) were IM immunized with 10^4^ HAD_50_ of the ASFV recombinant ΔMGF360/505_Stav strain. The reverse titration of the viral material for immunization confirmed that the calculated dose of 10^4^ HAD_50_ was used to inoculate the animals. Piglets in group 2 (*n* = 3), as well as animals in group 3 (*n* = 2), were inoculated with PBS.

In contrast to previous experiments, in this experiment, 3 out of 5 animals immunized with the ASFV recombinant ΔMGF360/505_Stav strain developed a low-grade fever lasting 1 day at 3–5 dpi (Supplementary Materials, Figure S4C, Table S4). No other clinical signs were observed (Supplementary Materials, Figure S4D, Table S4). In addition, in this experiment, ASFV DNA was detected in four blood samples of the ΔMGF360/505_Stav-inoculated pigs, collected at 7 dpi (## 1.1, 1.2, 1.3, 1.5) (2.24 × 10^3^–8.58 × 10^3^ the ASFV genome copies/mL) (Supplementary Materials, Table S4). All other blood samples were negative (Supplementary Materials, Table S4). No animals from any of the other groups demonstrated fever or any other clinical signs and viremia (Supplementary Materials, Figure S4, Table S4).

**Figure 5:**
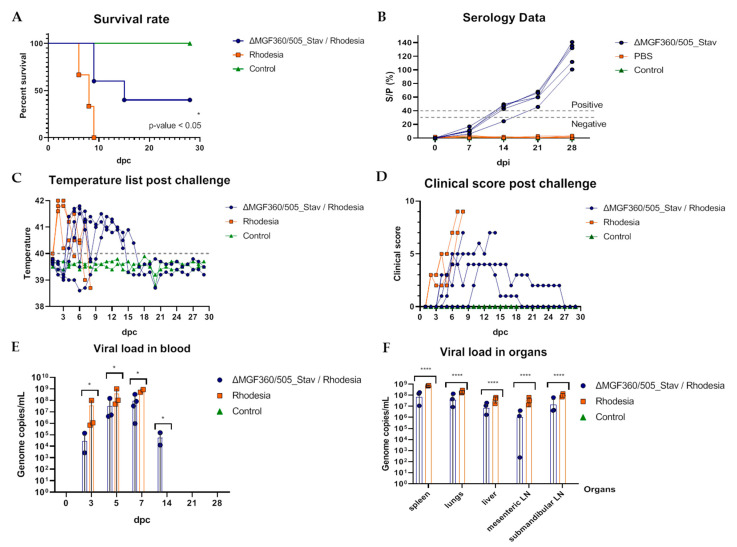
The efficiency of immunization with the ASFV recombinant ΔMGF360/505_Stav strain against the ASFV virulent Rhodesia strain, when pigs were immunized with 10^4^ HAD_50_ per animal and then challenged with 10^3^ HAD_50_ per animal of the ASFV heterologous Rhodesia strain (Experiment 3). The lethality kinetics (**A**), humoral immune response (**B**), body temperatures post-challenge (**C**), clinical signs post-challenge (**D**), daily viral loads in blood post-challenge (**E**), and post-mortem viral loads in organs (**F**) in pigs immunized with the ASFV recombinant ΔMGF360/505_Stav strain, infected with the Rhodesia strain (blue line), non-immunized animals, infected with the Rhodesia strain (orange line), and control non-inoculated animals (green line). (**B**) The dashed lines represent the cut-off value, above which values were considered positive, below which were considered negative, and between them, there was a gray area where the values were considered doubtful. (**C**) The fever cutoff (40 °C) is marked as a gray dashed line. Data on body temperature, clinical assessment, antibody response, and viral load are presented as individual values for each animal. dpi—days post-immunization; dpc—days post-challenge. * *p* < 0.05; **** *p* < 0.0001.

We did not observe significant differences in the kinetic of anti-ASFV antibody formation compared to previous experiments. Thus, all serum samples collected at 21–28 dpi and most sera at 14 dpi (except #1.3) were positive (Figure 5B, Supplementary Materials, Tables S4 and S5). In the remaining sera samples, antibodies against ASFV were not detected (Figure 5B, Supplementary Materials, Table S4).

ELISPOT assay results showed that PBMCs from all pigs immunized with the recombinant strain produced IFN-γ after stimulation by the parental strain, and also after stimulation by Concanavalin A (positive control). The numbers of spot-forming cells from immunized pigs were significantly higher than the cells from non-immunized pigs (Supplementary Materials, Figure S5B, Table S4). The numbers of spot-forming cells from immunized pigs stimulated with medium (negative control) were comparable with the numbers of spot-forming cells from non-immunized pigs (Supplementary Materials, Figure S5B, Table S4).

At 28 days post-inoculation, all piglets in group 1 and group 2 (positive control) were challenged intramuscularly with 10^3^ HAD_50_ per pig of the Rhodesia strain. Animals in group 3 (negative control) remained uninfected throughout the experiment.

Since the Rhodesia strain is also highly virulent, the mortality rate in group 2 (positive control) expectedly reached 100% by day 9 pc (Figure 5A, Table S4). The incubation period was estimated as 5–7 days. The onset of fever in animals of this group was noted at 2 dpc (Figure 5C, Table S4). A higher maximum clinical score (9) was characteristic of animals challenged with the Rhodesia strain (Figure 5D) compared to animals infected with the Stavropol_01/08 strain (5) (Experiment 2, Figure 4D).

We noted that the efficacy of the ASFV recombinant ΔMGF360/505_Stav strain against the heterologous Rhodesia strain was significantly lower than that observed in Experiment 2, amounting to 40% (Figure 5A, Supplementary Materials, Table S5). In group 2, two of the five animals survived until the conclusion of the experiment. Both surviving pigs exhibited prolonged periods of fever, lasting 7 to 9 days, in addition to other clinical signs, including apathy, feed refusal, and neurological disorders (Figure 5A,C,D). Regarding viremia in these surviving animals, ASFV DNA (1.51 × 10^5^ − 9.48 × 10^5^ the ASFV genome copies/mL) was detectable in blood at 7 dpc (#1.4) and 14 dpc (#1.1), while at later times, the genome was not detected (Figure 5E, Supplementary Materials, Tables S4 and S5). In contrast, significantly higher genome copy numbers (3.25 × 10^7^ to 3.3 × 10^8^ the ASFV genome copies/mL) were detected in the blood of the deceased animals in group 1, with a peak occurring at 7 days post-infection (dpc) (Figure 5E, Supplementary Materials, Tables S4 and S5).

It is noteworthy that the highest viral load values in the blood of infected animals from control group 2 were observed prior to their demise, occurring between 5 and 7 dpc, and ranged from 5.06 × 10^8^ to 1.01 × 10^9^ ASFV genome copies/mL (Figure 5E, Supplementary Materials, Tables S4 and S5). High viral load was characteristic of all examined organs of the control animals of group 2 (1.3 × 10^7^–8.04 × 10^8^ the ASFV genome copies/mL), as well as for three immunized animals that died after the ASF acute form (2.4 × 10^2^–1.82 × 10^8^ the ASFV genome copies/mL) (Figure 5F, Supplementary Materials, Tables S4 and S5). In contrast, we did not detect the viral genome in the lung, liver, spleen, and mesenteric and submandibular lymph nodes of two surviving immunized piglets. However, additional examination of the tissues of the joints, intestines, and tonsils of these animals revealed ASFV DNA only in the tonsils of animal #1.1 (2.17 × 10^5^ the ASFV genome copies/mL) (Supplementary Materials, Table S4).

No clinical signs, as well as the ASFV genome in the blood and organs, were noted in uninfected animals.

## 4. Discussion

Since the introduction of ASFV into the Russian Federation, the virus has spread widely to the western and eastern regions, causing outbreaks of the disease among both domestic pigs and wild boars [23,24]. To date, two LAVs against ASF are licensed in Vietnam and available in some Asian countries, but so far there are no globally approved vaccines. In Russia, there are also no developed or registered vaccines against ASF genotype II, although several vaccine strains against ASF of other genotypes/serotypes have been obtained [12,13]. Thus, the development of safe and effective vaccines against circulating and emerging ASFV variants is required.

The LAVs, predominantly based on recombinant viruses with deletions of virulence-associated genes, are considered the most promising vaccine candidates against ASF. Although many recombinant attenuated strains have been produced recently, not all of them have been characterized in detail. A number of well-characterized strains, including HLJ/18-7GD, ASFV-G-ΔI177L, and ASFV-G-ΔMGF, have demonstrated the capacity to induce complete protection against homologous virulent ASFV challenges under laboratory conditions [25,26,27,28,31,56]. Furthermore, these strains have been evaluated in field trials. However, the results of using these vaccines in the field are currently not completely clear.

The genomes of naturally low virulent strains such as NHV68 and OURT88, as well as strains attenuated by adaptation to cultured cell lines (BA71V and KK262), contain large fragmental deletions in the left variable region [46,57,58,59]. These large deletions include the genes MGF360-12L, MGF360-13L, MGF360-14L, MGF505-1R, MGF505-2R, and MGF505-3R, although they are not always limited to these genes. As already noted, deletions of these six genes of the MGF360 and MGF505 multigene families have been repeatedly used to create LAVs [25,27,31,60,61,62,63,64,65]. Importantly, these proteins mediate viral virulence through modulation of the host immune response [66,67] but do not affect the induction of a protective immune response.

Due to these facts, we decided to also use the deletion of these genes for the production of LAV against ASFV genotype 2 isolates circulating in Russia. We used the Russian ASFV Stavropol_01/08 strain (genotype II, serotype 8) as a backbone to generate a recombinant virus. Comparative complete genome analysis of the recombinant ΔMGF360/505_Stav and the parental Stavropol_01/08 strains confirmed the deletion of the multigene family MGF360/505 genes and the simultaneous insertion of the EGFP gene with the E184L gene promoter. In addition, we found few point mutations (insertions and deletions) in the poly-C and poly-G regions. Due to the fact that the mutations in the poly(C) and poly(G) regions are difficult to confirm by both Illumina and Sanger sequencing, we cannot say that these mutations are related to the recombinant strain production and are not sequencing errors.

To characterize the recombinant ΔMGF360/505_Stav strain, we conducted three animal experiments. The first animal experiment has confirmed that the recombinant ΔMGF360/505_Stav strain is fully attenuated, without any side effects during the observation period (24–28 dpi). Animals developed low levels of viremia in the early stages (3–7 dpi). As expected, anti-ASF antibodies were detected in animals starting from 14–21 dpi, and the kinetics of antibody formation were comparable with our other immunization/challenge experiments [43,46].

The efficacy of the new recombinant ΔMGF360/505_Stav strain against the parental Stavropol_01/08 strain was assessed in the second experiment. Considering that the strain we obtained was genetically very close (but not identical) to the ASFV-G-ΔMGF [25] and HLJ/18-6GD [31] strains, we assumed a high level of protection of ΔMGF360/505_Stav-immunized animals from infection with the homologous strain. As expected, 100% of ΔMGF360/505_Stav-immunized animals were completely protected from the homologous infection. Interestingly, in our experiment, we did not detect the genome of the ASFV virulent and the ASFV recombinant strains in the blood and organs post-challenge, although the virus was detected by other researchers [25,31].

It was previously shown that viruses within the serotype appear to cross-protect against one another while viruses outside the serotype do not [11,12]; thus, ASF protective immunity may be serotype-specific. However, these results were obtained using only vaccine strains attenuated by adaptation to cultured cell lines for immunization, while recombinant deletion variants were not used. In addition, the FRCVM, where the classification into serotypes (serogroups) was proposed, had not previously conducted work on the study of serotype 8 strain cross-immunity. Therefore, the aim of the third animal experiment was to study the cross-protection of ΔMGF360/505_Stav-immunized animals against a challenge with the ASFV heterologous Rhodesia, a serotype 8 strain. Previously, the Rhodesia strain was characterized as virulent (with a dose of 10^3^ HAD_50_) and was assigned to serotype 8 based on HAI typing and CD2v/lectin genotyping [13]. Based on the results of p72 genotyping, the Rhodesia strain was assigned to genotype VIII, while the Stavropol_01/08 strain was identified as genotype II [14].

In Experiment 3, we did not observe significant differences in the development of cellular or humoral immune responses in pigs after immunization with the ASFV recombinant ΔMGF360/505_Stav strain compared to the Experiment 2 data. However, only 40% of the ΔMGF360/505_Stav-immunized pigs were protected against challenge with the ASFV Rhodesia strain. Three immunized animals died at 9–15 dpc, and two immunized animals had a prolonged fever and other clinical signs but remained alive until the end of the observation period. Thus, the recombinant ΔMGF360/505_Stav strain cannot be used to protect against this heterologous variant of serotype 8.

The molecular and immunological foundations of cross-immunity remain poorly understood. However, further investigation into these aspects is crucial for the advancement of safe and effective vaccines against ASF. It has been previously demonstrated that although genotype I and II strains often had partial cross-immunity [29,36,38,40,41,45], ASF vaccines effective against ASFV genotype II do not protect against the highly virulent genotype I and II recombinant ASFV [20,56]. We plan to conduct a comparative analysis of the complete genomes of the Stavropol_01/08 and the Rhodesia strains and to continue the study of cross-immunity within ASF serotypes for a more detailed understanding of this important issue.

The small number of animals used in the experiments, as well as the lack of reversion-to-virulence and genetic stability studies, seem to be the main limitations of this research. Further experiments are required to confirm the safety and efficacy of this ASFV vaccine strain.

## 5. Conclusions

In this study, the obtained homologous recombination strain with a six-gene deletion in the MGF360/505 region located in the left variable region was characterized in vivo and in vitro. The genetic makeup of the recombinant ΔMGF360/505_Stav strain closely resembled the previously described ASFV-G-ΔMGF and HLJ/18-6GD strains. Our studies demonstrated that the recombinant ΔMGF360/505_Stav strain is fully attenuated, characterized by a short period of viremia, and it protected 100% of immunized animals against the homologous virulent strain. At the same time, only partial protection (40%) of the ΔMGF360/505_Stav-immunized pigs against challenge with the ASFV heterologous Rhodesia strain was shown. Therefore, we propose the recombinant ΔMGF360/505_Stav strain as the basis of a promising vaccine against a homologous ASFV genotype II. However, this LAV cannot be used to protect animals against heterologous variants of serotype 8.

## Data Availability

The complete genome sequences of the ASFV Stavropol_01/08 strain and the ASFV recombinant ΔMGF360/505_Stav strain were deposited in GenBank with accession numbers PQ672299 and PQ672300, respectively.

## Supplementary Materials

The following supporting information can be downloaded at: https://www.mdpi.com/article/10.3390/vaccines12121443/s1, Figure S1: Schemes of three in vivo experiments; Figure S2: Specific amplification of the recombination site and the reporter EGFP gene of the ASFV recombinant ΔMGF360/505_Stav strain or the recombination cassette by PCR; Figure S3: In vitro replication of the ASFV recombinant ΔMGF360/505_Stav strain in the porcine macrophages; Figure S4: Body temperature and ASF-specific clinical signs in the pigs post-immunization with the ASFV recombinant ΔMGF360/505_Stav strain and challenge with ASFV Stavropol_01/08 strain (Experiment 2) and post-immunization with the ASFV recombinant ΔMGF360/505_Stav strain and challenge with the ASFV Rhodesia strain (Experiment 3); Figure S5: Cellular response of immunized with the ASFV recombinant ΔMGF360/505_Stav strain pigs; Table S1: Results of the ASFV recombinant ΔMGF360/505_Stav strain serotyping; Table S2: The results of the study of the safety, immunogenicity, and in vivo replication of the ASFV recombinant ΔMGF360/505_Stav strain (Experiment 1); Table S3: The data of clinical signs, day of death, immune response, and viral load in blood and organs in animals in groups 1 and 2 (Experiment 2); Table S4: The data of clinical signs, day of death, immune response, and viral load in blood and organs in animals in groups 1 and 2 (Experiment 3); Table S5: Summary of the results of a comparative analysis of the effectiveness of the ASFV recombinant ΔMGF360/505_Stav strain to protect animals from infection with the ASFV homologous virulent parental Stavropol_01/08 strain or the ASFV heterologous virulent Rhodesia strain (Experiment 2 and 3).

## References

[1] Eustace Montgomery R. (1921). On a Form of Swine Fever Occurring in British East Africa (Kenya Colony). J. Comp. Pathol. Ther..

[2] Coggins L. (1974). African swine fever virus. Pathogenesis. Prog. Med. Virol..

[3] Mebus C.A. (1988). African swine fever. Adv. Virus Res..

[4] Dixon L.K., Chapman D.A., Netherton C.L., Upton C. (2013). African swine fever virus replication and genomics. Virus Res..

[5] Bastos A.D., Penrith M.L., Crucière C., Edrich J.L., Hutchings G., Roger F., Couacy-Hymann E., Thomson G.R. (2003). Genotyping field strains of African swine fever virus by partial p72 gene characterisation. Arch. Virol..

[6] Quembo C.J., Jori F., Vosloo W., Heath L. (2018). Genetic characterization of African swine fever virus isolates from soft ticks at the wildlife/domestic interface in Mozambique and identification of a novel genotype. Transbound. Emerg. Dis..

[7] Spinard E., Rai A., Osei-Bonsu J., O’Donnell V., Ababio P.T., Tawiah-Yingar D., Arthur D., Baah D., Ramirez-Medina E., Espinoza N. (2023). The 2022 Outbreaks of African Swine Fever Virus Demonstrate the First Report of Genotype II in Ghana. Viruses.

[8] Dinhobl M., Spinard E., Tesler N., Birtley H., Signore A., Ambagala A., Masembe C., Borca M.V., Gladue D.P. (2024). Reclassification of ASFV into 7 Biotypes Using Unsupervised Machine Learning. Viruses.

[9] Spinard E., Dinhobl M., Fenster J., Davis C., Borca M.V., Gladue D.P. (2024). Analysis of the Unique Historical Isolate of African Swine Fever Virus Isolate Spencer from Outbreaks in 1951. Viruses.

[10] Sereda A.D., Balyshev V.M. (2011). Antigenic diversity of African swine fever viruses. Vopr. Virusol..

[11] Vishnjakov I.F., Mitin N.I., Petrov J.I. (1995). Seroimmunological classification of African swine fever virus natural isolates. Topical Issues of Veterinary Virology. Proceedings of the Conference VNIIVViM: Classical Swine Fever Urgent Problems of Science and Practice.

[12] Sereda A.D., Balyshev V.M., Kazakova A.S., Imatdinov A.R., Kolbasov D.V. (2020). Protective Properties of Attenuated Strains of African Swine Fever Virus Belonging to Seroimmunotypes I–VIII. Pathogens.

[13] Malogolovkin A., Burmakina G., Tulman E.R., Delhon G., Diel D.G., Salnikov N., Kutish G.F., Kolbasov D., Rock D.L. (2015). African swine fever virus CD2v and C-type lectin gene loci mediate serological specificity. J. Gen. Virol..

[14] Malogolovkin A., Burmakina G., Titov I., Sereda A., Gogin A., Baryshnikova E., Kolbasov D. (2015). Comparative analysis of African swine fever virus genotypes and serogroups. Emerg. Infect. Dis..

[15] Thomson G.R. (1985). The epidemiology of African swine fever: The role of free-living hosts in Africa. Onderstepoort J. Vet. Res..

[16] Jori F., Vial L., Penrith M.L., Pérez-Sánchez R., Etter E., Albina E., Michaud V., Roger F. (2013). Review of the sylvatic cycle of African swine fever in sub-Saharan Africa and the Indian ocean. Virus. Res..

[17] Rowlands R.J., Michaud V., Heath L., Hutchings G., Oura C., Vosloo W., Dwarka R., Onashvili T., Albina E., Dixon L.K. (2008). African swine fever virus isolate, Georgia, 2007. Emerg. Infect. Dis..

[18] World Organisation for Animal Health: Home—WOAH. https://www.woah.org/en/disease/african-swine-fever/#ui-id-2.

[19] Sun E., Huang L., Zhang X., Zhang J., Shen D., Zhang Z., Wang Z., Huo H., Wang W., Huangfu H. (2021). Genotype I African swine fever viruses emerged in domestic pigs in China and caused chronic infection. Emerg. Microbes Infect..

[20] Zhao D., Sun E., Huang L., Ding L., Zhu Y., Zhang J., Shen D., Zhang X., Zhang Z., Ren T. (2023). Highly lethal genotype I and II recombinant African swine fever viruses detected in pigs. Nat. Commun..

[21] Le V.P., Nguyen V.T., Le T.B., Mai N.T.A., Nguyen V.D., Than T.T., Lai T.N.H., Cho K.H., Hong S.K., Kim Y.H. (2024). Detection of Recombinant African Swine Fever Virus Strains of p72 Genotypes I and II in Domestic Pigs, Vietnam, 2023. Emerg. Infect. Dis..

[22] Lee K., Hang Vu T.T., Yeom M., Nguyen V.D., Than T.T., Nguyen V.T., Jeong D.G., Ambagala A., Le V.P., Song D. (2024). Molecular Characterization of Emerging Recombinant African Swine Fever Virus of Genotype I and II in Vietnam, 2023. Emerg. Microbes Infect..

[23] Gogin A., Gerasimov V., Malogolovkin A., Kolbasov D. (2013). African swine fever in the North Caucasus region and the Russian Federation in years 2007–2012. Virus Res..

[24] Kolbasov D., Titov I., Tsybanov S., Gogin A., Malogolovkin A. (2018). African Swine Fever Virus, Siberia, Russia, 2017. Emerg. Infect. Dis..

[25] O’Donnell V., Holinka L.G., Gladue D.P., Sanford B., Krug P.W., Lu X., Arzt J., Reese B., Carrillo C., Risatti G.R. (2015). African Swine Fever Virus Georgia Isolate Harboring Deletions of MGF360 and MGF505 Genes Is Attenuated in Swine and Confers Protection against Challenge with Virulent Parental Virus. J. Virol..

[26] Ta H.L. (2022). Quality Control of a Live—Attenuated African Swine Fever Vaccine in Viet Nam.

[27] Borca M.V., Ramirez-Medina E., Silva E., Vuono E., Rai A., Pruitt S., Holinka L.G., Velazquez-Salinas L., Zhu J., Gladue D.P. (2020). Development of a Highly Effective African Swine Fever Virus Vaccine by Deletion of the I177L Gene Results in Sterile Immunity against the Current Epidemic Eurasia Strain. J. Virol..

[28] Tran X.H., Le T.T.P., Nguyen Q.H., Do T.T., Nguyen V.D., Gay C.G., Borca M.V., Gladue D.P. (2022). African swine fever virus vaccine candidate ASFV-G-∆I177L efficiently protects European and native pig breeds against circulating Vietnamese field strain. Transbound. Emerg. Dis..

[29] Gallardo C., Sanchez E.G., Perez-Nunez D., Nogal M., de Leon P., Carrascosa A.L., Nieto R., Soler A., Arias M.L., Revilla Y. (2018). African swine fever virus (ASFV) protection mediated by NH/P68 and NH/P68 recombinant live-attenuated viruses. Vaccine.

[30] O’Donnell V., Risatti G.R., Holinka L.G., Krug P.W., Carlson J., Velazquez-Salinas L., Azzinaro P.A., Gladue D.P., Borca M.V. (2017). Simultaneous Deletion of the 9GL and UK Genes from the African Swine Fever Virus Georgia 2007 Isolate Offers Increased Safety and Protection against Homologous Challenge. J. Virol..

[31] Chen W., Zhao D., He X., Liu R., Wang Z., Zhang X., Li F., Shan D., Chen H., Zhang J. (2020). A seven-gene-deleted African swine fever virus is safe and effective as a live attenuated vaccine in pigs. Sci. China Life Sci..

[32] Reis A.L., Abrams C.C., Goatley L.C., Netherton C., Chapman D.G., Sanchez-Cordon P., Dixon L.K. (2016). Deletion of African swine fever virus interferon inhibitors from the genome of a virulent isolate reduces virulence in domestic pigs and induces a protective response. Vaccine.

[33] Reis A.L., Goatley L.C., Jabbar T., Sanchez-Cordon P.J., Netherton C.L., Chapman D.A.G., Dixon L.K. (2017). Deletion of the African Swine Fever Virus Gene DP148R Does Not Reduce Virus Replication in Culture but Reduces Virus Virulence in Pigs and Induces High Levels of Protection against Challenge. J. Virol..

[34] Gladue D.P., Ramirez-Medina E., Vuono E., Silva E., Rai A., Pruitt S., Espinoza N., Velazquez-Salinas L., Borca M.V. (2021). Deletion of the A137R Gene from the Pandemic Strain of African Swine Fever Virus Attenuates the Strain and Offers Protection against the Virulent Pandemic Virus. J. Virol..

[35] Perez-Nunez D., Sunwoo S.Y., Garcia-Belmonte R., Kim C., Vigara-Astillero G., Riera E., Kim D.M., Jeong J., Tark D., Ko Y.S. (2022). Recombinant African Swine Fever Virus Arm/07/CBM/c2 Lacking CD2v and A238L Is Attenuated and Protects Pigs against Virulent Korean Paju Strain. Vaccines.

[36] Monteagudo P.L., Lacasta A., López E., Bosch L., Collado J., Pina-Pedrero S., Correa-Fiz F., Accensi F., Navas M.J., Vidal E. (2017). BA71DCD2: A new recombinant live attenuated African swine fever virus with cross-protective capabilities. J. Virol..

[37] Zhang J., Zhang Y., Chen T., Yang J., Yue H., Wang L., Zhou X., Qi Y., Han X., Ke J. (2021). Deletion of the L7L-L11L genes attenuates ASFV and induces protection against homologous challenge. Viruses.

[38] Leitao A., Cartaxeiro C., Coelho R., Cruz B., Parkhouse R.M.E., Portugal R., Vigario J.D., Martins C.L.V. (2001). The non-haemadsorbing African swine fever virus isolate ASFV/NH/P68 provides a model for defining the protective anti-virus im-mune response. J. Gen. Virol..

[39] King K., Chapman D., Argilaguet J.M., Fishbourne E., Hutet E., Cariolet R., Hutchings G., Oura C.A.L., Netherton C.L., Moffat K. (2011). Protection of European domestic pigs from virulent African isolates of African swine fever virus by experimental immunisation. Vaccine.

[40] Sanchez-Cordon P.J., Chapman D.A.G., Jabbar T., Reis A.L., Goatley L.C., Netherton C.L., Taylor G., Montoya M., Dixon L.K. (2017). Different routes and doses influence protection in pigs immunised with the naturally attenuated African swine fever virus isolate OURT88/3. Antivir. Res..

[41] Vlasov M., Sindryakova I., Kudryashov D., Morgunov S., Kolbasova O., Lyska V., Zhivoderov S., Pivova E., Balyshev V., Namsrayn S. (2024). Administration Routes and Doses of the Attenuated African Swine Fever Virus Strain PSA-1NH Influence Cross-Protection of Pigs against Heterologous Challenge. Animals.

[42] Krug P.W., Holinka L.G., O’Donnell V., Reese B., Sanford B., Fernandez-Sainz I., Gladue D.P., Arzt J., Rodriguez L., Risatti G.R. (2015). The progressive adaptation of a Georgian isolate of African swine fever virus to Vero cells leads to a gradual attenuation of virulence in swine corresponding to major modifications of the viral genome. J. Virol..

[43] Titov I., Burmakina G., Morgunov Y., Morgunov S., Koltsov A., Malogolovkin A., Kolbasov D. (2017). Virulent strain of African swine fever virus eclipses its attenuated derivative after challenge. Arch. Virol..

[44] Sereda A.D., Vlasov M.E., Koltsova G.S., Morgunov S.Y., Kudrjashov D.A., Sindryakova I.P., Kolbasova O.L., Lyska V.M., Koltsov A.Y., Zhivoderov S.P. (2022). Immunobiological Characteristics of the Attenuated African Swine Fever Virus Strain Katanga-350. Viruses.

[45] Vlasov M.E., Sindryakova I.P., Kudrjashov D.A., Morgunov S.Y., Kolbasova O.L., Lyska V.M., Zhivoderov S.P., Pivova E.Y., Balyshev V.M., Sereda A.D. (2023). Inoculation with ASFV-Katanga-350 Partially Protects Pigs from Death during Sub-sequent Infection with Heterologous Type ASFV-Stavropol 01/08. Viruses.

[46] Kholod N., Koltsov A., Krutko S., Tulman E.R., Namsrayn S., Kutish G.F., Belov S., Korotin A., Sukher M., Koltsova G. (2023). Comparison of Attenuated and Virulent Strains of African Swine Fever Virus Genotype I and Serogroup 2. Viruses.

[47] Koltsov A., Sukher M., Krutko S., Belov S., Korotin A., Rudakova S., Morgunov S., Koltsova G. (2024). Towards Safe African Swine Fever Vaccines: The A137R Gene as a Tool to Reduce Virulence and a Promising Serological DIVA Marker Candidate. Animals.

[48] Reed L.J., Muench H. (1938). A simple method of estimating fifty per cent endpoints. Am. J. Epidemiol..

[49] Burmakina G., Malogolovkin A., Tulman E.R., Zsak L., Delhon G., Diel D.G., Shobogorov N.M., Morgunov Y.P., Morgunov S.Y., Kutish G.F. (2016). African swine fever virus serotype-specific proteins are significant protective antigens for African swine fever. J. Gen. Virol..

[50] Ambagala A., Goonewardene K., Kanoa I.E., Than T.T., Nguyen V.T., Lai T.N.H., Nguyen T.L., Erdelyan C.N.G., Robert E., Tailor N. (2024). Characterization of an African Swine Fever Virus Field Isolate from Vietnam with Deletions in the Left Variable Multigene Family Region. Viruses.

[51] Koltsova G., Koltsov A., Krutko S., Kholod N., Tulman E.R., Kolbasov D. (2021). Growth kinetics and protective efficacy of attenuated ASFV strain Congo with deletion of the EP402 gene. Viruses.

[52] Howey E.B., O’Donnell V., de Carvalho Ferreira H.C., Borca M.V., Arzt J. (2013). Pathogenesis of highly virulent African swine fever virus in domestic pigs exposed via intraoropharyngeal, intranasopharyngeal, and intramuscular inoculation, and by direct contact with infected pigs. Virus Res..

[53] Toussaint J.F., Sailleau C., Breard E., Zientara S., De Clercq K. (2007). Bluetongue virus detection by two real-time RT-qPCRs targeting two different genomic segments. J. Virol. Methods.

[54] Petrov A., Beer M., Blome S. (2014). Development and validation of a harmonized TaqMan-based triplex real-time RT-PCR protocol for the quantitative detection of normalized gene expression profiles of seven porcine cytokines. PLoS ONE.

[55] Burmakina G., Malogolovkin A., Tulman E.R., Xu W., Delhon G., Kolbasov D., Rock D.L. (2019). Identification of T-cell epitopes in African swine fever virus CD2v and C-type lectin proteins. J. Gen. Virol..

[56] Diep N.V., Duc N.V., Ngoc N.T., Dang V.X., Tiep T.N., Nguyen V.D., Than T.T., Maydaniuk D., Goonewardene K., Ambagala A. (2024). Genotype II Live-Attenuated ASFV Vaccine Strains Unable to Completely Protect Pigs against the Emerging Recombinant ASFV Genotype I/II Strain in Vietnam. Vaccines.

[57] Portugal R., Coelho J., Höper D., Little N.S., Smithson C., Upton C., Martins C., Leitão A., Keil G.M. (2015). Related strains of African swine fever virus with different virulence: Genome comparison and analysis. J. Gen. Virol..

[58] Rodríguez J.M., Moreno L.T., Alejo A., Lacasta A., Rodríguez F., Salas M.L. (2015). Genome sequence of African swine fever virus BA71, the virulent parental strain of the nonpathogenic and tissue-culture adapted BA71V. PLoS ONE.

[59] Bao Y.-J., Qiu J., Rodríguez F., Qiu H.-J. (2020). The Genetic Variation Landscape of African Swine Fever Virus Reveals Frequent Positive Selection on Amino Acid Replacements. bioRxiv.

[60] Kitamura T., Masujin K., Yamazoe R., Kameyama K.-i., Watanabe M., Ikezawa M., Yamada M., Kokuho T. (2023). A Spontaneously Occurring African Swine Fever Virus with 11 Gene Deletions Partially Protects Pigs Challenged with the Parental Strain. Viruses.

[61] Bourry O., Hutet E., Le Dimna M., Lucas P., Blanchard Y., Chastagner A., Paboeuf F., Le Potier M.-F. (2022). Oronasal or Intramuscular Immunization with a Thermo-Attenuated ASFV Strain Provides Full Clinical Protection against Georgia 2007/1 Challenge. Viruses.

[62] Deutschmann P., Forth J.H., Sehl-Ewert J., Carrau T., Viaplana E., Mancera J.C., Urniza A., Beer M. (2023). Assessment of African swine fever vaccine candidate ASFV-G-∆MGF in a reversion to virulence study. npj Vaccines.

[63] Rathakrishnan A., Connell S., Petrovan V., Moffat K., Goatley L.C., Jabbar T., Sánchez-Cordón P.J., Reis A.L., Dixon L.K. (2022). Differential Effect of Deleting Members of African Swine Fever Virus Multigene Families 360 and 505 from the Genotype II Georgia 2007/1 Isolate on Virus Replication, Virulence, and Induction of Protection. J. Virol..

[64] Deutschmann P., Carrau T., Sehl-Ewert J., Forth J.H., Viaplana E., Mancera J.C., Urniza A., Beer M., Blome S. (2022). Taking a Promising Vaccine Candidate Further: Efficacy of ASFV-G-ΔMGF after Intramuscular Vaccination of Domestic Pigs and Oral Vaccination of Wild Boar. Pathogens.

[65] Xie Z., Liu Y., Di D., Liu J., Gong L., Chen Z., Li Y., Yu W., Lv L., Zhong Q. (2022). Protection Evaluation of a Five-Gene-Deleted African Swine Fever Virus Vaccine Candidate Against Homologous Challenge. Front. Microbiol..

[66] Gao Q., Yang Y., Quan W., Zheng J., Luo Y., Wang H., Chen X., Huang Z., Chen X., Xu R. (2021). The African Swine Fever Virus with MGF360 and MGF505 Deleted Reduces the Apoptosis of Porcine Alveolar Macrophages by Inhibiting the NF-κB Signaling Pathway and Interleukin-1β. Vaccines.

[67] Sunwoo S.-Y., García-Belmonte R., Walczak M., Vigara-Astillero G., Kim D.-M., Szymankiewicz K., Kochanowski M., Liu L., Tark D., Podgórska K. (2024). Deletion of MGF505-2R Gene Activates the cGAS-STING Pathway Leading to Attenuation and Protection against Virulent African Swine Fever Virus. Vaccines.

