# Supplementary material for: Construction of the First Russian Recombinant Live Attenuated Vaccine Strain and Evaluation of Its Protection Efficacy Against Two African Swine Fever Virus Heterologous Strains of Serotype 8
Source: Vaccines (Basel). 2024 Dec 21;12(12):1443. doi: 10.3390/vaccines12121443 (PMC11680325; doi:10.3390/vaccines12121443)
Supplement: Supplementary file 1 [file vaccines-12-01443-s001.zip › vaccines-3293627-supplementary.pdf]

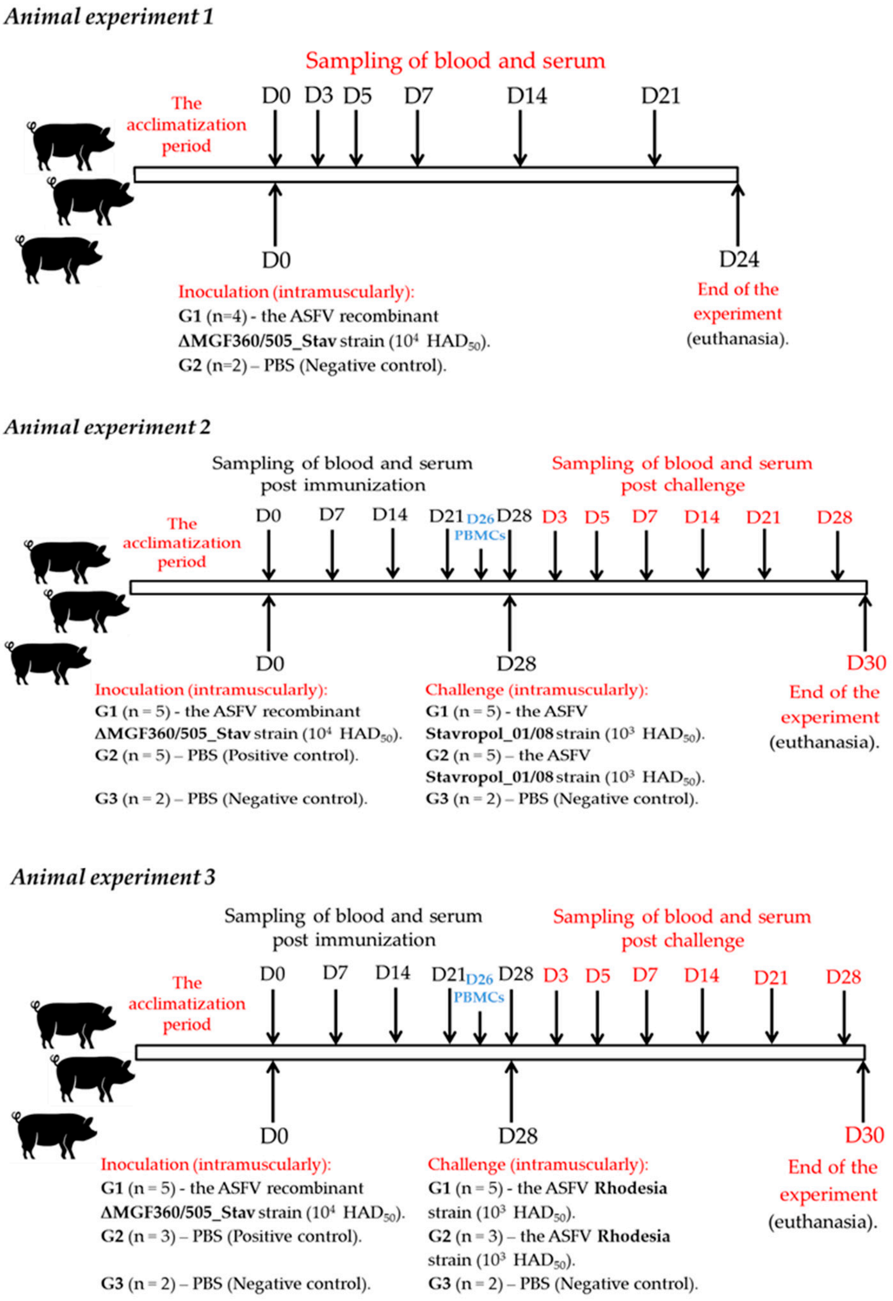

Figure S1. Schemes of three *in vivo* experiments. (A) Experiment 1 consisted of experimental procedures for the inoculation with 10^4 HAD_{50} of the ASFV recombinant ΔMGF360/505_Stav strain (Group 1) or PBS (Group 2) and sample collection. The inoculation was performed at 0 dpi, and blood and sera samples were collected at 0, 3, 5, 7, 14, and 21 dpi. The observation period was 24 dpi. (B) Experiment 2 consisted of experimental procedures for the inoculation with 10^4 HAD_{50} of the ASFV recombinant ΔMGF360/505_Stav strain (Group 1) or PBS (Groups 2 and 3), a challenge with 10^3 HAD_{50} of the ASFV virulent Stavropol_01/08 strain (Groups 1 and 2), and sample collection. The inoculation was performed at 0 dpi, the challenge was performed at 28 dpi, blood and serum samples were collected at 0, 7, 14, 21 and 28 dpi, as well as 3, 5, 7, 14, 21 and 28 dpc. Whole peripheral blood (with heparin) was collected at 26 dpi. The observation period was 30 dpc. (C) Experiment 3 consisted of experimental procedures for the inoculation with 10^4 HAD_{50} of the ASFV recombinant ΔMGF360/505_Stav strain (Group 1) or PBS (Group 2 and 3), the challenge with 10^3 HAD_{50} of the ASFV virulent Rhodesia strain (Group 1 and 2) and sample collection. The inoculation was performed at 0 dpi, the challenge was performed at 28 dpi, blood and serum samples were collected at 0, 7, 14, 21 and 28 dpi, as well as 3, 5, 7, 14, 21 and 28 dpc. Whole peripheral blood (with heparin) was collected at 26 dpi. The observation period was 28 dpc.

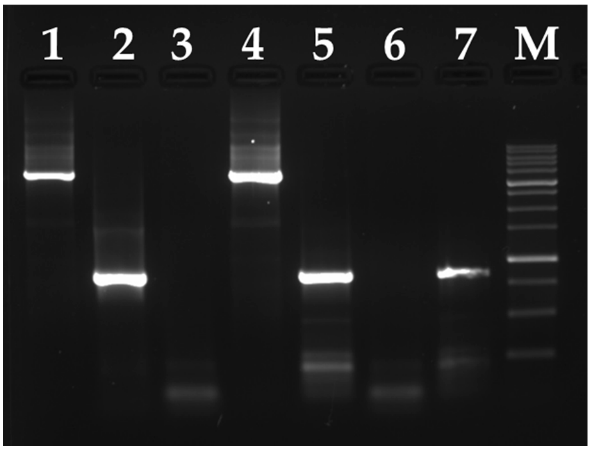

Figure S2. Specific amplification of the recombination site and the reporter EGFP gene of the ASFV recombinant ΔMGF360/505_Stav strain or the recombination cassette by PCR. The recombination site was amplified using primers DelMGF360/505_Larm_EcoRI_F and DelMGF360/505_Rarm_SphI_R. The specific PCR fragments were analyzed by electrophoresis in 1.5% agarose gel: lane 1 shows the PCR fragment of the complete recombination site of the ASFV recombinant ΔMGF360/505_Stav strain; lane 2 shows the PCR fragment of the reporter EGFP gene with the E184L gene promoter of the ASFV recombinant ΔMGF360/505_Stav strain; lane 4 shows the PCR fragment of the complete recombination site of the recombination cassette; lane 5 shows the PCR fragment of the reporter EGFP gene with the E184L gene promoter of the recombination cassette; lane 3 and 6 show negative controls; lane 7 shows positive control (the pUC57 vector with EGFP gene with the E184L gene promoter); lane M contains a 1 kb DNA ladder (Evrogen, Russia).

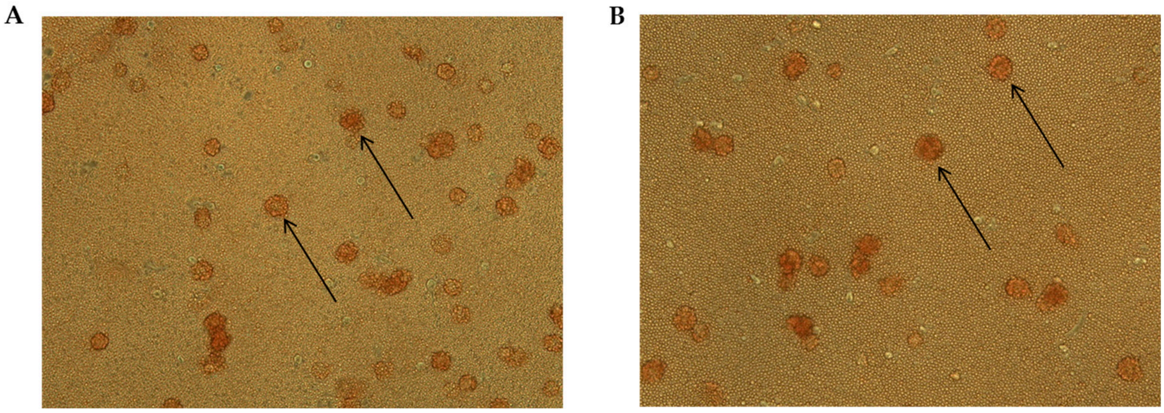

Figure S3. *In vitro* replication of the ASFV recombinant ΔMGF360/505_Stav strain in the porcine macrophages. Hemadsorption of cells infected with the ASFV parental Stavropol/01/08 strain (A) or the ASFV recombinant ΔMGF360/505_Stav strain (B). The arrows point to the erythrocyte rosettes around ASFV-infected cells (A,B).

Table S1. Results of the ASFV recombinant ΔMGF360/505_Stav strain serotyping using the ASFV parental Stavropol_01/08 strain, reference viruses and sera (serotype 1 (SG1), serotype 2 (SG2), serotype 3 (SG3), serotype 4 (SG4), serotype 8 (SG8)) by hemadsorption inhibition assays.

**ASFV strain**	**HAI titer***					
	**Anti-ASFV reference serum**					**Negative serum**
	**SG1**	**SG2**	**SG3**	**SG4**	**SG8**	
L57 (SG1)	+ 1:16	-	-	-	-	-
K49 (SG2)	-	+ 1:32	-	-	-	-
M78 (SG3)	-		+ 1:32	-	-	-
F32 (SG4)	-			+ 1:16		-
Rhodesia (SG8)	-				+ 1:32	-
Stavropol_01/08 (SG8)	-				+ 1:16-1:32	-
ΔMGF360/505_Stav	-	-	-	-	+ 1:16-1:32	-

SG – serotype (serogroup) based on HAI assay results.
*Dilution of the serogroup-specific reference serum resulting in complete HAI. The results are representative of two or more independent experiments.

Table S2. The results of the study of safety, immunogenicity and *in vivo* replication of the ASFV recombinant ΔMGF360/505_Stav strain (Experiment 1).

**Experiment/ Group**	**N of animals**	**Mortality**		**Fever**		**Serology**		**Viral load in blood (Min-Max)** genome copies/mL	**Viral load in organs (Max)** genome copies/mL
		%	TTD	%	TTF	ELISA %	HAI %		
Experiment 1 **ΔMGF360/505_Stav**	**4**	**0**	**-**	**0**	**-**	**100**	**0**	**3-7 dpi** 1,3e+002-5,11e+005	**Neg**
Experiment 1 **Control Group**	**2**	**0**	**-**	**0**	**-**	**0**	**0**	**0**	**Neg**

TTD, Mean time-to-death in days post-challenge, with SE in parentheses.
TTF, Mean time-to-fever in days post-challenge, with SE in parentheses.

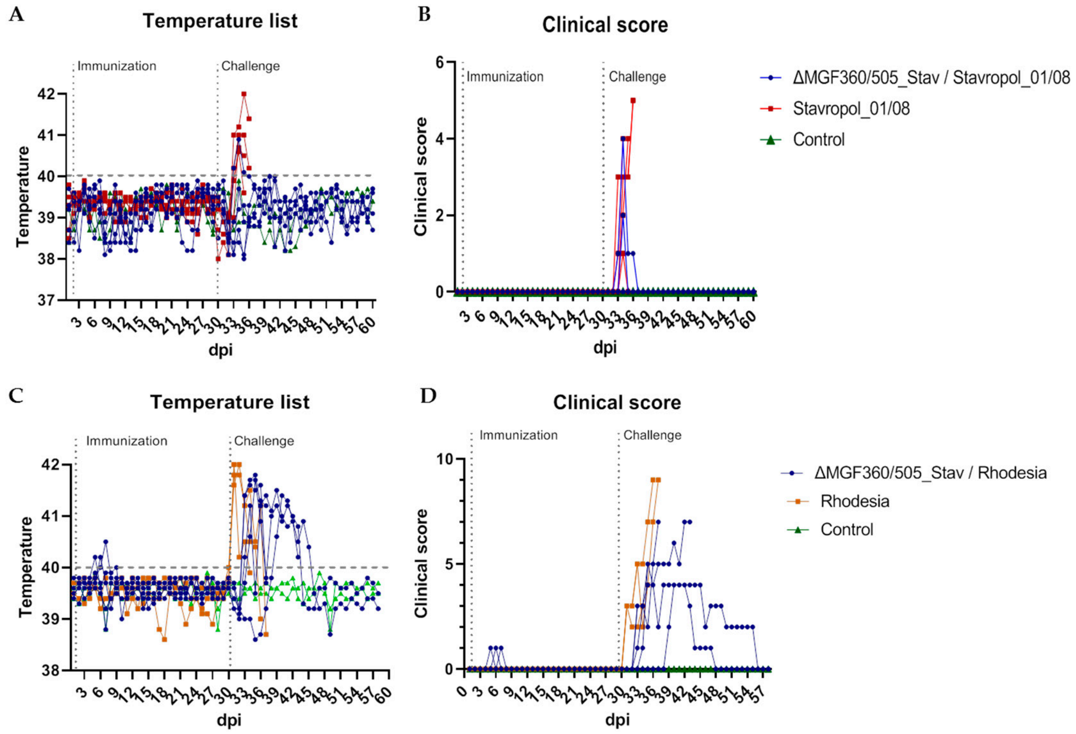

Figure S4. Body temperature and ASF-specific clinical signs in pigs post immunization with the ASFV recombinant ΔMGF360/505_Stav strain and challenge with ASFV Stavropol_01/08 strain (Experiment 2) and post immunization with the ASFV recombinant ΔMGF360/505_Stav strain and challenge with the ASFV Rhodesia strain (Experiment 3). Experiment 2 - Body temperatures (A), clinical signs (B) in pigs immunized with the ASFV recombinant ΔMGF360/505_Stav strain, challenged with the Stavropol_01/08 strain (blue line), non-immunized animals, infected with the Stavropol_01/08 strain (red line), and control non-inoculated animals (green line). Experiment 3 - Body temperatures (C), clinical signs (D) in pigs immunized with the ASFV recombinant ΔMGF360/505_Stav strain, challenged with the Rhodesia strain (blue line), non-immunized animals, infected with the Rhodesia strain (orange line), and control non-inoculated animals (green line). Data on body temperature and clinical assessment presented as individual values for each animal.

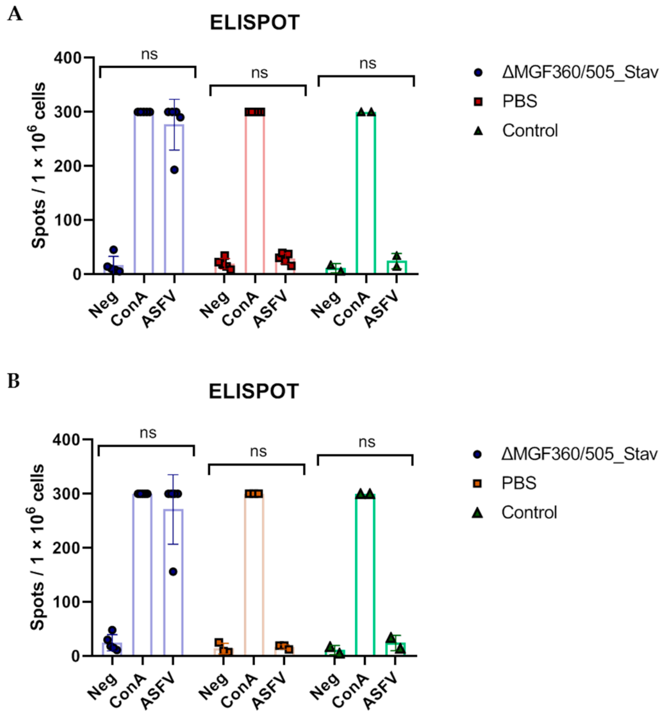

Figure S5. Cellular response of immunized with the ASFV recombinant ΔMGF360/505_Stav strain pigs. ELISPOT testing the ASFV-specific IFN-γ-producing PBMCs collected at 26 dpi in the Experiment 2 (A) or the Experiment 3 (B). The PBMCs were stimulated with ASFV antigen (ASFV, the ASFV Stavropol_01/08 strain), Concanavalin A (ConA, positive control), medium (Neg, negative control). The data are presented as the mean ± standard deviation (SD).

Table S3. The data of clinical signs, day of dead, immune response, viral load in blood and organs in animals in group1 and 2 (Experiment 2).

**# animal**	**ASFV strain**	**Pre-Challenge Fever**	**Post-Challenge Fever**	**Pre-Challenge clinical signs**	**Post-Challenge clinical signs**	**Pre-Challenge ELISA**	**Pre-Challenge ELISPOT** (Spots / 1×10^6 cells)	**Pre-Challenge viremia** (Min-Max, genome copies/mL)	**Post-Challenge viremia** (Min-Max, genome copies/mL)	**Viral load in organs** (Min-Max, genome copies/mL)	**Day of dead**
**1/1**		**No**	**No**	**No**	**4 dpc**	**Pos**	**Pos** (>300)	**Neg**	**Neg**	**Neg**	**30 dpc***
**1/2**	**ΔMGF360/505_Stav / Stavropol_01/08**	**No**	**3-5 dpc**	**No**	**3-5 dpc**	**Pos**	**Pos** (>300)	**Neg**	**Neg**	**Neg**	**30 dpc***
**1/3**		**No**	**No**	**No**	**No**	**Pos**	**Pos** (290)	**Neg**	**Neg**	**Neg**	**30 dpc***
**1/4**		**No**	**No**	**No**	**No**	**Pos**	**Pos** (193)	**Neg**	**Neg**	**Neg**	**30 dpc***
**1/5**		**No**	**No**	**No**	**No**	**Pos**	**Pos** (>300)	**Neg**	**Neg**	**Neg**	**30 dpc***
**2/1**		**No**	**3-4 dpc**	**No**	**3-4 dpc**	**Neg**	**Neg**	**Neg**	**3 dpc** (3,22e+007)	3,86e+007-6,33e+008	**5 dpc**
**2/2**		**No**	**4-5 dpc**	**No**	**4-5 dpc**	**Neg**	**Neg**	**Neg**	**3-5 dpc** (9,57e+007 4,73e+008)	2,4e+007-5,59e+008	**6 dpc**
**2/3**	**Stavropol_01/08**	**No**	**4-5 dpc**	**No**	**4-5 dpc**	**Neg**	**Neg**	**Neg**	**3-5 dpc** (1,62e+008 3,89e+008)	2,06e+006-9,46e+007	**6 dpc**
**2/4**		**No**	**4-6 dpc**	**No**	**4-6 dpc**	**Neg**	**Neg**	**Neg**	**3-5 dpc** (8,19e+005 2,3e+008)	4,8e+006-1,21e+008	**7 dpc**
**2/5**		**No**	**3-6 dpc**	**No**	**3-6 dpc**	**Neg**	**Neg**	**Neg**	**3-5 dpc** (7,48e+007 4,61e+008)	9,2e+006-2,23e+008	**7 dpc**

* - The animals were humanely euthanized after the end of the observation period.

No - Not observed.

Neg - Negative test result.

Pos - Positive test result.

Table S4. The data of clinical signs, day of dead, immune response, viral load in blood and organs in animals in group1 and 2 (Experiment 3).

# animal	ASFV strain	Pre-Challenge Fever	Post-Challenge Fever	Pre-Challenge clinical signs	Post-Challenge clinical signs	Pre-Challenge ELISA	Pre-Challenge ELISPOT (Spots / 1×10^6 cells)	Pre-Challenge viremia (Min-Max, genome copies/mL)	Post-Challenge viremia (Min-Max, genome copies/mL)	Viral load in organs (Min-Max, genome copies/mL)	Day of dead
**1/1**		**No**	**10-16 dpc**	**No**	**10-26 dpc**	**Pos**	**Pos** (>300)	**7-dpi** 3,18e+003	**14 dpc** (1,51e+005)	Only tonsils (2,17E+05)	**28 dpc***
**1/2**		**No**	**4-8 dpc**	**No**	**4-8 dpc**	**Pos**	**Pos** (156)	**7 dpi** 8,58e+003	**3-7 dpc** (2,68e+003-3,25e+007)	4,13e+006-1,41e+008	**9 dpc**
**1/3**	**ΔMGF360/505_Stav / Rhodesia**	**4 dpi**	**4-8 dpc**	**4 dpi**	**4-8 dpc**	**Pos**	**Pos** (>300)	**7 dpi** 2,24e+003	**3-14 dpc** (1,27e+004-8,18e+007)	1,33e+006-1,82e+008	**9 dpc**
**1/4**		**5 dpi**	**5-14 dpc**	**5 dpi**	**5-18 dpc**	**Pos**	**Pos** (>300)	**Neg**	**7 dpc** (9,48e+005)	**Neg**	**28 dpc***
**1/5**		**3 dpi**	**4-14 dpc**	**3 dpi**	**4-14 dpc**	**Pos**	**Pos** (>300)	**7 dpi** 2,8e+003	**5-7 dpc** (1,47e+008-3,3e+008)	2,4e+002-1,13e+007	**15 dpc**
**2/1**		**No**	**2-8 dpc**	**No**	**2-8 dpc**	**Neg**	**Neg**	**Neg**	**3-7 dpc** (6,9e+005-5,06e+008)	1,3e+007-6,71e+008	**9 dpc**
**2/2**	**Rhodesia**	**No**	**2-5 dpc**	**No**	**2-5 dpc**	**Neg**	**Neg**	**Neg**	**3-5 dpc** (9,86e+007-1,01e+009)	1,73e+007-7,63e+008	**6 dpc**
**2/3**		**No**	**3-7 dpc**	**No**	**3-7 dpc**	**Neg**	**Neg**	**Neg**	**3-7 dpc** (1,11e+006-8,6e+008)	3,24e+007-8,04e+008	**8 dpc**

* - The animals were humanely euthanized after the end of the observation period.

No - Not observed.

Neg - Negative test result.

Pos - Positive test result.

Table S5. Summary of the results of a comparative analysis of the efficacy of the ASFV recombinant ΔMGF360/505_Stav strain to protect animals from infection with the ASFV homologous virulent parental Stavropol_01/08 strain or the ASFV heterologous virulent Rhodesia strain (Experiment 2 and 3).

**Experiment/ Group**	**N of animals**	**Mortality**		**Fever**		**Pre-challenge serology**		**Pre-challenge ELISPOT**	**Viral load in blood (Max)**	**Viral load in organs (Max)**
		%	TTD	%	TTF	ASFV ELISA %	HAI %	%	genome copies/mL	genome copies/mL
Experiment 2 **ΔMGF360/505_Stav/ Stavropol_01/08**	**5**	**0**	**-**	**20**	**3**	**100**	**0**	**100**	**0**	**0**
Experiment 2 **Stavropol_01/08**	**5**	**100**	**6.2**	**100**	**3.6**	**0**	**0**	**0**	**4,73e+008**	**6,33e+008**
Experiment 3 **ΔMGF360/505_Stav/ Rhodesia**	**5**	**60**	**11**	**100**	**5.4**	**100**	**0**	**100**	**3,3e+008**	**1,82e+008**
Experiment 3 **Rhodesia**	**3**	**100**	**7.67**	**100**	**2.33**	**0**	**0**	**0**	**1,01e+009**	**8,04e+008**
Experiment 2 **Control Group**	**2**	**0**	**-**	**0**	**-**	**0**	**0**	**0**	**0**	**0**
Experiment 3 **Control Group**	**2**	**0**	**-**	**0**	**-**	**0**	**0**	**0**	**0**	**0**

TTD, Mean time-to-death in days post-challenge, with SE in parentheses.
TTF, Mean time-to-fever in days post-challenge, with SE in parentheses.
